# The Effect of Adjuvant Therapy with Molecular Hydrogen on Endogenous Coenzyme Q_10_ Levels and Platelet Mitochondrial Bioenergetics in Patients with Non-Alcoholic Fatty Liver Disease

**DOI:** 10.3390/ijms241512477

**Published:** 2023-08-05

**Authors:** Zuzana Sumbalová, Jarmila Kucharská, Zuzana Rausová, Anna Gvozdjáková, Mária Szántová, Branislav Kura, Viliam Mojto, Ján Slezák

**Affiliations:** 1 Pharmacobiochemical Laboratory of 3rd Department of Internal Medicine, Faculty of Medicine, Comenius University in Bratislava, 811 08 Bratislava, Slovakia; jarmila.kucharska@fmed.uniba.sk (J.K.); zuzana.rausova@fmed.uniba.sk (Z.R.); anna.gvozdjakova@fmed.uniba.sk (A.G.); 2 3rd Department of Internal Medicine, Faculty of Medicine, Comenius University in Bratislava, 813 72 Bratislava, Slovakia; maria.szantova@kr.unb.sk (M.S.); viliam.mojto@kr.unb.sk (V.M.); 3 Institute for Heart Research, Center of Experimental Medicine, Slovak Academy of Sciences, 841 04 Bratislava, Slovakia; branislav.kura@savba.sk (B.K.); jan.slezak@savba.sk (J.S.)

**Keywords:** molecular hydrogen, platelets, mitochondria, oxidative phosphorylation, coenzyme Q_10_

## Abstract

Molecular hydrogen (H_2_) has been recognized as a novel medical gas with antioxidant and anti-inflammatory effects. Non-alcoholic fatty liver disease (NAFLD) is a liver pathology with increased fat accumulation in liver tissue caused by factors other than alcohol consumption. Platelet mitochondrial function is considered to reflect systemic mitochondrial health. We studied the effect of adjuvant therapy with hydrogen-rich water (HRW) on coenzyme Q_10_ (CoQ_10_) content and platelet mitochondrial bioenergetics in patients with NAFLD. A total of 30 patients with NAFLD and 15 healthy volunteers were included in this clinical trial. A total of 17 patients (H_2_ group) drank water three × 330 mL/day with tablets producing HRW (>4 mg/L H_2_) for 8 weeks, and 13 patients (P group) drank water with placebo tablets producing CO_2_. The concentration of CoQ_10-TOTAL_ was determined by the HPLC method, the parameter of oxidative stress, thiobarbituric acid reactive substances (TBARS), by the spectrophotometric method, and mitochondrial bioenergetics in platelets isolated from whole blood by high-resolution respirometry. The patients with NAFLD had lower concentrations of CoQ_10-TOTAL_ in the blood, plasma, and platelets vs. the control group. Mitochondrial CI-linked LEAK respiration was higher, and CI-linked oxidative phosphorylation (OXPHOS) and CII-linked electron transfer (ET) capacities were lower vs. the control group. Plasma TBARS concentrations were higher in the H_2_ group. After 8 weeks of adjuvant therapy with HRW, the concentration of CoQ_10_ in platelets increased, plasma TBARS decreased, and the efficiency of OXPHOS improved, while in the P group, the changes were non-significant. Long-term supplementation with HRW could be a promising strategy for the acceleration of health recovery in patients with NAFLD. The application of H_2_ appears to be a new treatment strategy for targeted therapy of mitochondrial disorders. Additional and longer-term studies are needed to confirm and elucidate the exact mechanisms of the mitochondria-targeted effects of H_2_ therapy in patients with NAFLD.

## 1. Introduction

Nonalcoholic fatty liver disease (NAFLD) is a metabolic dysfunction of the liver characterized by excessive fat accumulation in the liver tissue. It is defined by the presence of lipid droplets (steatosis) in more than 5% of hepatocytes in the absence of other etiologies of liver disease [1,2]. NAFLD is the most common chronic liver disease, affecting around 25% of the world’s population. It has been recognized as a hepatic manifestation of the metabolic syndrome associated with overweight, obesity, and insulin resistance. Recently, a new term, metabolic (dysfunction)-associated fatty liver disease (MAFLD), was suggested, emphasizing the important role of metabolic dysfunction in its pathogenesis [2]. NAFLD can progress to nonalcoholic steatohepatitis (NASH), an inflammatory condition associated with liver fibrosis [1], and eventually progress to cirrhosis associated with portal hypertension, end-stage liver disease requiring liver transplantation, or hepatocellular carcinoma [3]. Patients with NAFLD have a higher risk of atherosclerosis and cardiovascular diseases [4].

The following sequence of metabolic alterations leads to NAFLD: The excessive intake of calories leads to the accumulation of fat in adipose tissue in the form of triglycerides. The hypertrophied adipocytes secrete more pro-inflammatory adipokines. Excessive reactive oxygen species (ROS) produced by the mitochondrial respiratory chain during increased oxidation of fat and the infiltration of macrophages secreting pro-inflammatory mediators contribute to chronic inflammation in adipose tissue and the development of insulin resistance (IR) [5]. IR in the adipose tissue leads to lipolysis, resulting in increased free fatty acids in plasma. IR of peripheral tissues results in chronic hyperglycemia and hyperinsulinemia, which stimulate de novo lipogenesis in the liver. In NAFLD, hepatic fat accumulation originates from adipose tissue lipolysis (60%), hepatic de novo lipogenesis, and a high-calorie diet [6]. The accumulation of fat in hepatocytes in the form of triglycerides is a protective mechanism against toxic free fatty acids and is not hepatotoxic alone, but it increases the susceptibility to steatohepatitis and fibrosis.

Mitochondrial dysfunction and oxidative stress play a significant role in the development and progression of NAFLD, but the exact mechanisms are not fully understood [7,8]. Mitochondrial function and fatty acid oxidation (FAO) may increase during the development of NAFLD and gradually decrease in the progression of the disease [9,10]. The function of mitochondrial respiratory complexes may be affected by alterations in the lipid profile, as documented in mice liver mitochondria during NAFLD progression [11]. Decreased FAO, reduced mitochondrial function and ATP production, increased ROS production, increased outer mitochondrial membrane permeability, impaired mitochondrial protein synthesis, decreased mitochondrial biogenesis and mitophagy, and oxidative stress-mediated deletions of mtDNA have been reported in NAFLD [1,7,12,13]. In patients with NASH, ultrastructural changes in mitochondria (the loss of mitochondrial cristae and paracrystalline inclusions) were found in hepatic tissue [14]. In a mouse model of NAFLD, Krishnasamy et al. [15] showed the depolarization of mitochondria early in the course of NAFLD development, which was followed by increased mitophagy, suppressed mitochondrial biogenesis and dynamics, and mitochondrial depletion. Simoes et al. [10] showed the progressive decline of the liver mitochondrial function after the 20th week of Western diet-induced NAFLD in mice. Mitochondrial ROS production also declined with the progression of NAFLD, while the number of peroxisomes along with the upregulation of peroxisomal FAO. The authors concluded that the key players in the progression of NAFLD are not mitochondrial ROS but ROS generated during peroxisomal FAO or other cytosolic ROS [10].

Molecular hydrogen (H_2_) is the smallest antioxidant with a molecular weight of 2, which can easily penetrate into all organs and cell structures and reach subcellular compartments, such as mitochondria, ER, and nuclei. H_2_ is able to directly react with strong oxidants such as hydroxyl radical (·OH) and peroxynitrite (ONOO–) [16]. H_2_ also reduces oxidative stress indirectly by inducing antioxidant systems [17]. By modulating multiple signaling pathways and regulating various gene expressions, H_2_ can act as an anti-inflammatory and anti-apoptotic agent and a stimulator of energy metabolism [17,18,19]. H_2_ has been recognized as a novel medical gas [17], and beneficial effects of therapy with H_2_ have been documented in various diseases such as cardiovascular, neurodegenerative, inflammatory, kidney diseases, metabolic syndrome, diabetes, liver diseases, and cancer [20]. Multiple beneficial effects of treatment with hydrogen water have also been documented in plants. H_2_ can promote plant growth by regulating the effects of plant hormones such as auxin and cytokinin [21]. H_2_-based treatments ameliorate stress responses in plants, including salinity, heavy metals, temperature, and light stresses. Treatment with H_2_ could be prospectively used in agriculture to reduce postharvest senescence [22].

In a mouse model of NASH, hepatic mRNA expression of tumor necrosis factor-α (TNF-α), interleukin-6, fatty acid synthesis-related genes, and peroxisome proliferator-activated receptor-α decreased significantly after treatment with hydrogen-rich water (HRW), and the oxidative stress biomarker 8-hydroxydeoxyguanosine in the liver tissue was reduced [23]. In a high-fat diet-induced NAFLD rat model, treatment with H_2_ reduced body weight gain, improved glucose and lipid metabolism, attenuated hepatic steatosis, and improved hepatic mitochondrial dysfunction [24]. Treatment with HRW reduced liver fat accumulation in patients with NAFLD [25]. The effect of H_2_ administration on mitochondrial function, ADP production, and CoQ_10_ levels in patients with NAFLD has not been studied.

Platelets are an accessible source of mitochondria, and mitochondrial function in platelets is considered to be a marker of mitochondrial health [26]. The aim of our study was to analyze the effect of an 8-week administration of HRW to patients with NAFLD on platelet mitochondrial bioenergetics, endogenous levels of antioxidants coenzyme Q_10_ (CoQ_10_), α-tocopherol, β-carotene, and a parameter of oxidative stress, thiobarbituric acid reactive substances (TBARS). We tested the hypothesis that treatment with high-concentration HRW can improve mitochondrial function and CoQ_10_ levels in patients with NAFLD. This study was placebo-controlled and registered as a clinical trial.

## 2. Results

### 2.1. The Anthropometric and Biochemical Parameters in Groups of Patients with NAFLD

The patients with NAFLD had various stages of fibrosis, from stage F0 to stage F4. Accidentally, more patients with higher stages of fibrosis were included in the H_2_ group (F0(8), F1(1), F2(3), F3(2), and F4(3)) than in the P group (F0(4), F1(8), and F3(1)). Nevertheless, the difference between groups in the mean value of the fibrosis stage was statistically non-significant (1.47 ± 0.39 in the H_2_ group vs. 0.85 ± 0.22 in the P group, *p* = 0.21). All patients were on antihypertensive, antidiabetic, or hypolipidemic therapy with satisfactory control of blood pressure, blood glucose, blood lipids, and uric acid levels. The H_2_ and P groups did not differ in the incidence of hypertension, diabetes, or dyslipoproteinemia, and drug use was in similar proportions in both groups.

The anthropometric and biochemical parameters shown in Table 1 demonstrate that the patients with NAFLD were obese and had hyperglycemia and increased activities of the liver enzymes alanine transaminase (ALT), aspartate transaminase (AST), and gamma-glutamyl transferase (GMT). There was no difference between groups at baseline in any of the studied parameters (Table 1). After 8 weeks of treatment, the average value of the body mass index (BMI) did not change significantly in any group. However, the waist circumference decreased in the group of patients on adjuvant therapy with HRW by 1.9 cm (*p* = 0.038) (Table 1). In this group, the concentration of lymphocytes in the blood (the cells involved in the immune response) increased by 11.5% (*p* = 0.018), and another important parameter, the concentration of high-density lipoprotein (HDL) cholesterol, which was initially lower vs. the control group, increased by 7.2% (*p* = 0.00003) (Table 1). The concentration of albumin increased after the 8-week treatment in both groups of patients. The levels of total cholesterol, HDL cholesterol, and albumin were within the clinically normal range in all groups.

### 2.2. The Effect of Treatment with HRW on Platelet Mitochondrial Function

**Figure 1:**
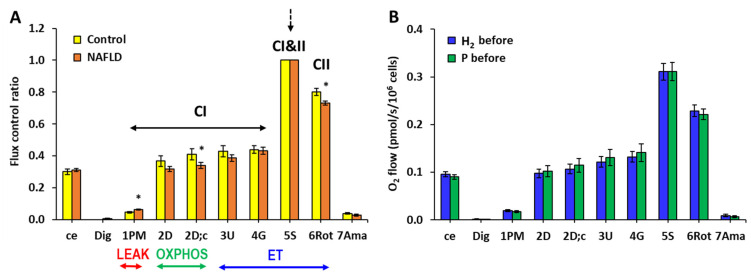
Bioenergetics in platelet mitochondria at the beginning of the study. (**A**) Parameters of mitochondrial respiration and ATP production in platelets of patients with NAFLD and the healthy volunteers expressed as flux control ratio. (**B**) Parameters of mitochondrial respiration and ATP production in platelets of the hydrogen (H_2_) and placebo (P) groups of patients with NAFLD at the beginning of the study expressed as O_2_ flow (pmol/s/10^6^ cells). The bars show mean ± standard error of mean (sem). The evaluated respiratory capacities are marked according to the titration steps in the substrate–uncoupler–inhibitor titration (SUIT) reference protocol 1 [27] and correspond to following respiratory states: ce—routine respiration of intact cells; Dig—residual oxygen consumption (ROX) after permeabilization with digitonin; 1PM—LEAK respiration with CI-linked substrates pyruvate and malate; 2D—CI-linked OXPHOS capacity (associated with ATP production); 2D; c—CI-linked OXPHOS capacity after addition of cytochrome *c* as a test for integrity of outer mitochondrial membrane; 3U—CI-linked electron transfer (ET) capacity with pyruvate and malate; 4G—CI-linked ET capacity with pyruvate, malate, and glutamate; 5S—CI&II-linked ET capacity; 6Rot—CII-linked ET capacity; 7Ama—ROX after inhibition of mitochondrial CIII. CI—respiration related to mitochondrial CI activity; CI&II—respiration related to mitochondrial CI and CII activity; CII—respiration related to mitochondrial CII activity. LEAK—non-phosphorylating state of respiration; OXPHOS—the capacity of oxidative phosphorylation; ET—the capacity of electron transfer. * *p* < 0.05 vs. the control group.

Mitochondrial respiration measured in isolated platelets in the whole group of patients with NAFLD was not significantly different from the respiration measured in the control group when evaluated as O_2_ flux per cells; however, internal normalization of the respiration revealed relatively higher CI-linked LEAK respiration and relatively lower CI-linked oxidative phosphorylation (OXPHOS) capacity and CII-linked ET capacity in the group of patients with NAFLD (Figure 1A). These differences vs. the control group indicate reprogramming of mitochondrial function in patients with NAFLD. There was no difference in the parameters of mitochondrial respiration in platelets between the H_2_ and P groups of patients at the beginning of the study (Figure 1B).

**Figure 2:**
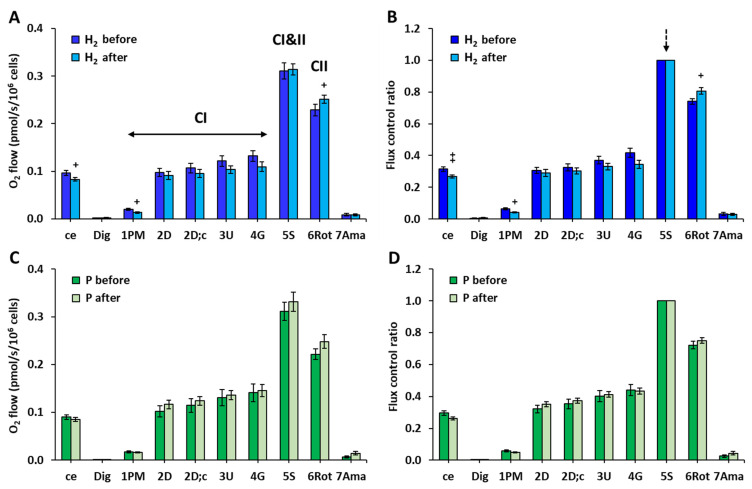
Bioenergetics in platelet mitochondria in the groups of NAFLD patients at the beginning and end of the study. (**A**,**C**) Parameters of mitochondrial respiration and ATP production in platelets of H_2_ and P groups of patients with NAFLD expressed as O_2_ flow (pmol/s/10^6^ cells). (**B**,**D**) Parameters of mitochondrial respiration and ATP production in platelets of H_2_ and P groups of patients with NAFLD expressed as flux control ratio. The bars show mean ± standard error of mean (sem). The evaluated respiratory capacities are marked according to the titration steps in the substrate–uncoupler–inhibitor titration (SUIT) reference protocol 1 [27] and correspond to following respiratory states: ce—routine respiration of intact cells; Dig—residual oxygen consumption (ROX) after permeabilization with digitonin; 1PM—LEAK respiration with CI-linked substrates pyruvate and malate; 2D—CI-linked OXPHOS capacity (associated with ATP production); 2D; c—CI-linked OXPHOS capacity after addition of cytochrome *c* as a test for integrity of outer mitochondrial membrane; 3U—CI-linked electron transfer (ET) capacity with pyruvate and malate; 4G—CI-linked ET capacity with pyruvate, malate, and glutamate; 5S—CI&II-linked ET capacity; 6Rot—CII-linked ET capacity; 7Ama—ROX after inhibition of mitochondrial CIII. H_2_ before, H_2_ after—the group of patients with NAFLD before and after 8-week adjuvant therapy with HRW; P before, P after—the group of patients with NAFLD before and after 8 weeks of taking placebo. CI—respiration related to mitochondrial CI activity; CI&II—respiration related to mitochondrial CI and CII activity; CII—respiration related to mitochondrial CII activity. + *p* < 0.05, ++ *p* < 0.01 vs. the same group at the beginning of the study.

After 8 weeks of treatment with HRW, routine respiration of intact cells and CI-linked LEAK respiration decreased and CII-linked ET capacity increased (Figure 2A). The internal normalization of each measurement for the maximum flux (5S—CI&II-linked ET capacity) confirmed these differences in the H_2_ group vs. baseline values (Figure 2B). There were no significant changes in the parameters of platelet mitochondrial respiration and ATP production after the 8-week treatment in the P group (Figure 2C,D).

**Figure 3:**
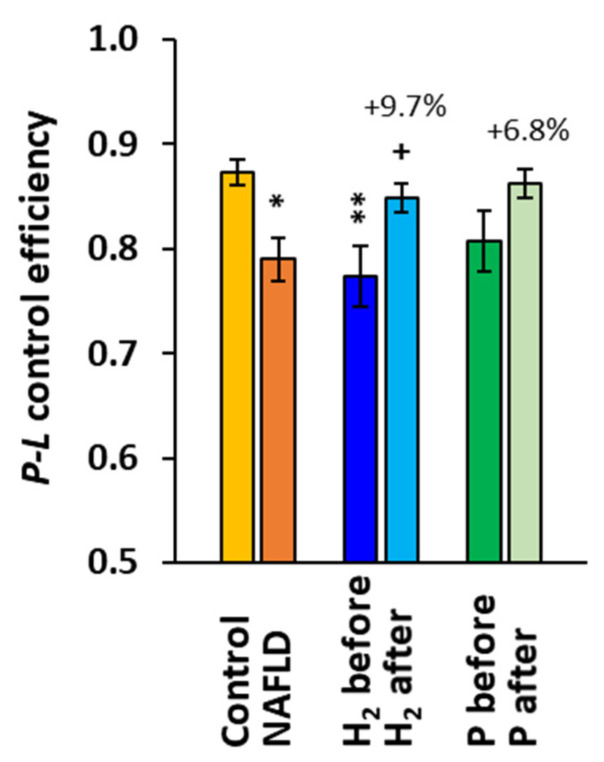
*P-L* control efficiency in platelet mitochondria in the control group, NAFLD patients, and groups H_2_ and P of NAFLD patients at the beginning and the end of the study. *P-L* control efficiency was calculated from parameters of mitochondrial respiration as (2D-1PM)/2D (for parameters of mitochondrial respiration refer to Figure 1). * *p* < 0.05, ** *p* < 0.01 vs. the control group; + *p* < 0.05 vs. the same group at the beginning of the study. The values (%) above the bars show the relative change vs. baseline values in each group.

The parameter *P-L* control efficiency was significantly reduced in the whole group of patients with NAFLD, reaching 90.5% (*p* = 0.013, Figure 3) of the control group values. In the H_2_ group, this parameter was significantly lower at baseline when compared to the control group (−11.4%, *p* = 0.007), and it increased by 9.7% (*p* = 0.043) after the 8-week treatment, while in the P group, the increase vs. baseline (+6.8%) was statistically non-significant (Figure 3).

### 2.3. The Effect of Treatment with HRW on Endogenous Coenzyme Q_10_ Concentration

**Figure 4:**
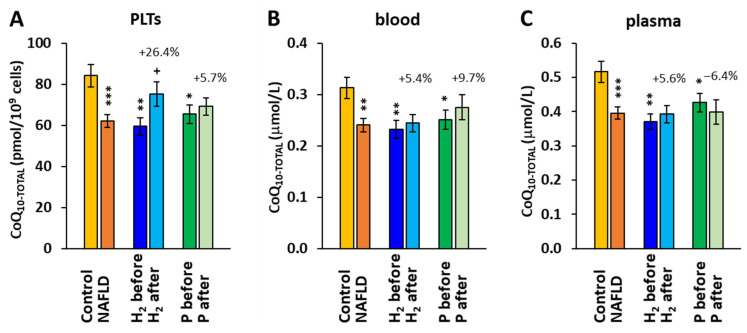
Coenzyme Q_10-TOTAL_ concentration in platelets (PLTs) (**A**), blood (**B**), and plasma (**C**). Control—the control group; H_2_ before, H_2_ after—the group of patients with NAFLD before and after 8-week adjuvant therapy with HRW; P before, P after—the group of patients with NAFLD before and after 8 weeks of taking placebo. * *p* < 0.05, ** *p* < 0.01, *** *p* < 0.001 vs. the control group; + *p* < 0.05 vs. the same group at the beginning of the study. The values (%) above the bars show the relative change vs. baseline values in each group.

The concentrations of CoQ_10_ in platelets (PLTs), blood, and plasma were, at the beginning of the study, reduced in the whole group of patients with NAFLD (−26.3%, *p* < 0.001) and in both groups H_2_ (−29.3, *p* = 0.0015) and P (−22.3%, *p* = 0.016) of patients vs. the control group. After the 8-week therapy, the concentration of CoQ_10_ in the H_2_ group increased in PLTs (+26.4% vs. the H_2_ before, *p* = 0.012) (Figure 4A). In blood and plasma, the concentrations of CoQ_10_ did not significantly change after the 8-week treatment in any group.

**Figure 5:**
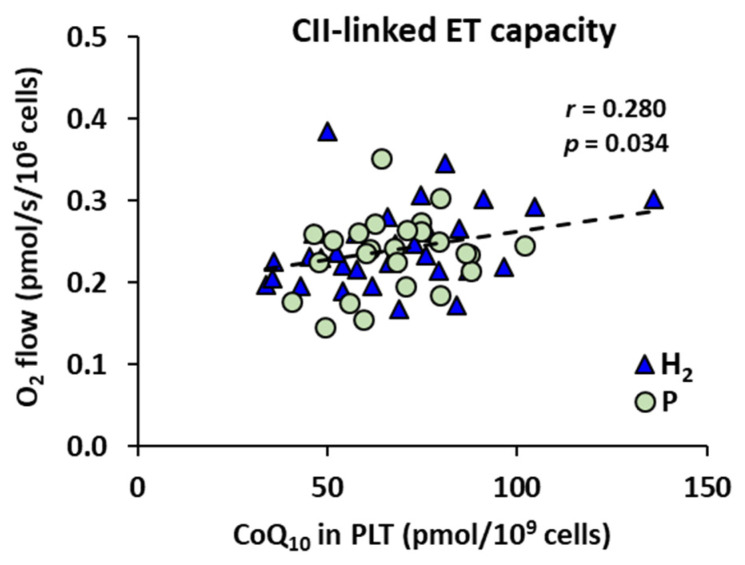
The correlation between CII-linked ET capacity and concentration of CoQ_10_ in platelets. H_2_—the group of patients with NAFLD before and after adjuvant treatment with HRW; P—the group of patients with NAFLD before and after treatment with placebo; *r*—Pearson correlation coefficient; *p*—the *p*-value.

Mitochondrial CII-linked ET capacity in platelets of patients with NAFLD correlated with the concentration of CoQ_10_ in platelets (*r* = 0.280, *p* = 0.034) (Figure 5), when calculated for all measurements performed with platelets of NAFLD patients (*n* = 57), showing dependence of mitochondrial respiration on CoQ_10_ content. The groups H_2_ and P are marked for more information.

### 2.4. The Effect of Treatment with HRW on α-tocopherol, β-carotene, and TBARS Concentrations

**Figure 6:**
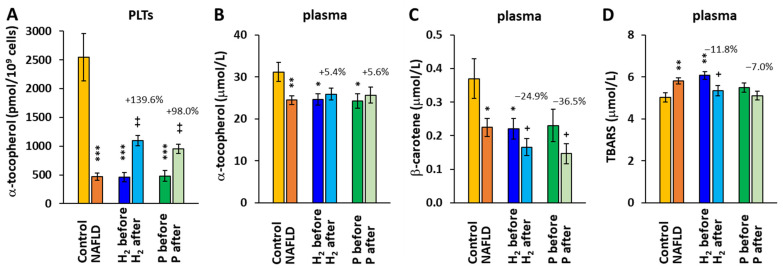
The concentration α-tocopherol in platelets (**A**) and plasma (**B**); the concentration of β-ca-rotene in plasma (**C**); and the concentration of thiobarbituric acid reactive substances (TBARS) in plasma (**D**). Control—the control group; H_2_ before, H_2_ after—the group of patients with NAFLD before and after 8-week adjuvant therapy with HRW; P before, P after—the group of patients with NAFLD before and after 8 weeks of taking placebo. * *p* < 0.05, ** *p* < 0.01, *** *p* < 0.001 vs. the control group; + *p* < 0.05, ++ *p* < 0.01 vs. the same group at the beginning of the study. The values (%) above the bars show the relative change vs. baseline values in each group.

The concentration of α-tocopherol in platelets and plasma was significantly reduced at baseline in the whole group of patients with NAFLD and both H_2_ and P groups vs. the control group (Figure 6A,B). After the 8-week therapy, α-tocopherol concentrations in platelets increased in both the H_2_ and P groups by 139.6% (*p* = 0.005) and 98.0% (*p* = 0.004), respectively (Figure 6A). The increase in the α-tocopherol concentration in plasma was not statistically significant, and its values were within the clinically normal range in all the groups.

The concentration of β-carotene in the plasma of patients with NAFLD was significantly reduced vs. the control group values at baseline, and it further decreased in both groups after the 8-week treatment (−24.9%, *p* = 0.032 in the H_2_ group and −36.5%, *p* = 0.037 in the P group) (Figure 6C).

The parameter of lipid peroxidation—plasma TBARS—was increased in the H_2_ group at baseline, and after 8-week adjuvant therapy with HRW, it decreased (−11.8%, *p* = 0.025) (Figure 6D).

## 3. Discussion

In this placebo-controlled clinical trial, we studied the effect of adjuvant therapy with HRW in patients with NAFLD treated for this diagnosis for a longer period of time. Our patients with NAFLD had various stages of fibrosis, from stage F0 to stage F4. In the group on adjuvant treatment with HRW, more patients with higher stages of fibrosis were included than in the P group, but the difference between groups in the mean value of the fibrosis stage was statistically non-significant. There was no statistically significant difference between groups at baseline in any of the studied parameters. After the 8-week treatment, the waist circumference decreased by 1.9 cm (*p* = 0.038) in patients drinking HRW, while in the placebo group, the decrease was statistically non-significant. Drinking HRW improved the lipid profile, as documented by the increased concentration of HDL cholesterol (+7.2%, *p* = 0.0003). An improvement was also observed in the concentration of immune cells (lymphocytes) in the peripheral blood (+11.5%, *p* = 0.018).

An increase in HDL cholesterol and decrease in urinary TBARS were reported in patients with potential metabolic syndrome who drank H_2_ water for 12 weeks [28]. In patients with metabolic syndrome, the blood glucose and cholesterol levels significantly declined, and the biomarkers of inflammation improved after 24 weeks of supplementation with high-concentration HRW [29]. In these patients, the waist-to-hip circumference ratio and body mass index declined, and many other parameters such as total cholesterol, HDL cholesterol, very low-density lipoprotein (VLDL) cholesterol, triglycerides, and heart rate improved [29]. We did not find changes in blood glucose levels after the 8-week adjuvant treatment of patients with NAFLD with HRW of similar H_2_ concentration. Overall, the treatment with HRW affected very few biochemical and anthropometrical parameters (Table 1) and had no significant effect on the levels of the inflammatory markers TNF-α, nuclear factor kappa B [30]. The discrepancy with the results of the study of LeBaron et al. [29] could come from the much shorter duration of the adjuvant treatment with HRW in our study, pointing to the need for longer-term studies.

In our study, we determined mitochondrial function by respirometric measurements in isolated platelets from patients with NAFLD. We have shown that mitochondrial metabolism in patients with NAFLD was reprogrammed with relatively higher CI-linked LEAK respiration, lower CI-linked OXPHOS capacity, lower CII-linked ET capacity, and lower *P-L* coupling efficiency. The impairment of mitochondrial function in liver mitochondria has been reported in animal and human studies. The respiration in liver mitochondria of obese people without NAFLD was elevated vs. the controls and declined gradually with the severity of NAFLD, keeping higher rates than in the healthy controls [9]. On the other hand, Pérez-Carreras et al. [31] reported significantly decreased activities of all complexes (CI—CV) of the respiratory chain in the liver tissue of patients with NAFLD. FAO, mitochondrial biogenesis, mitophagy, and dynamics in hepatocytes declined with the increased severity of NAFLD in patients [13]. The respiratory control ratio was reduced in the liver mitochondria of obese people and declined even more with NAFLD progression [9].

We studied mitochondrial bioenergetics in platelets, which may reflect systemic mitochondrial health. The *P-L* control efficiency as a more correct (from the statistical reason [32]) alternative of the respiratory control ratio was reduced in patients with NAFLD (Figure 3), which is similar to the study of Koliaki et al. [9].

The alterations in mitochondrial bioenergetics in platelets in patients with NAFLD were diminished after the 8-week adjuvant treatment with HRW. Similarly, the CoQ_10_ concentration in platelets was reduced in patients with NAFLD at the beginning of the study and has improved after the adjuvant therapy with HRW. CoQ_10_ is an integral part of the mitochondrial electron transport chain. It is involved in the process of oxidative phosphorylation. CoQ_10_ serves as a mobile electron and proton carrier, transferring electrons from CI, CII, and the electron-transferring flavoprotein complex to CIII. CoQ_10_ is present in oxidized form (ubiquinone), reduced form (ubiquinol), and semiquinone radical intermediates formed at the catalytic centers of CI and CIII, which are the main sources of mitochondrial ROS. The correlation between the CoQ_10-TOTAL_ concentration in platelets and CII-linked ET capacity (Figure 5) shows that this parameter of mitochondrial respiration depends on the CoQ_10_ content, and the deficit of CoQ_10_ may negatively affect CII-linked respiration and ATP production.

The stimulating effect of H_2_ water on energy metabolism has been reported previously. In a db/db obesity mice model, drinking H_2_ water for 3 months stimulated energy metabolism (evaluated by increased O_2_ consumption and CO_2_ production in calorimetric measurements), decreased oxidative stress in the liver, significantly reduced neutral lipid accumulation in the liver, and suppressed body weight gain [33]. In our previous study, the administration of HRW to control rats increased CI- and CII-linked ADP-stimulated respiration in heart mitochondria and the concentration of CoQ_9_ in the heart tissue and mitochondria, and reduced the parameter of oxidative stress—malondialdehyde in plasma [34,35].

In a sepsis model in mice, the inhalation of 2% hydrogen gas prevented lung injury by modulating mitochondrial function and dynamics [36] and decreased brain injury by inducing mitochondrial biogenesis [37]. In the rat model of sepsis-associated encephalopathy (induced by lipopolysaccharide, LPS), intraperitoneal administration of hydrogen-rich saline 1 h after LPS administration improved survival rate, and attenuated neuroinflammation, neuronal injury, and mitochondrial dysfunction [38].

The results of the present study support the evidence that mitochondria may be one of the key targets of H_2_ therapy. The mechanism of the effect of H_2_ in mitochondria is not fully understood. H_2_ affects mitochondrial bioenergetics indirectly through gene-expression alterations [33,39,40]. The direct effects of H_2_ on the mitochondrial electron transfer chain are investigated. It has been proposed that H_2_ may function as both an electron and proton donor in the Q cycle and convert the quinone intermediates to the fully reduced ubiquinol, thereby increasing the antioxidant capacity of the quinone pool and preventing ROS generation [34,41]. H_2_ may react with highly reactive and unstable semiquinone forms: SQ_Nf_ in complex I and semiquinone at the Q_o_-site in complex III [41]. The possibility of H_2_ activation in the quinone-binding space (Q-chamber) of the catalytic center of CI was proposed [41], and suppressed superoxide generation at CI in the presence of H_2_ was measured in vitro [42]. H_2_ was able to alter the direction of electron flow from reversed electron transfer (RET) to forward electron transfer (FET) in in vitro experiments and reduce the mitochondrial membrane potential in vivo [42]. The recent discovery of Fe-porphyrin as a primary molecular target/biosensor of H_2_ has enabled a deeper understanding of the biomedical effects of H_2_ [43,44]. It has been shown that the oxidized Fe-porphyrin in a free or protein-bound form can catalyze the hydrogenation and scavenging of ROS by H_2_, especially the hydroxyl radical (·OH) [43]. Mitochondria isolated from A549 cells consumed nearly equal amounts of H_2_ from the hydrogen-rich saline than the whole cells, confirming mitochondria as the main intracellular target of H_2_ [43]. The highest concentration of Fe-porphyrin is in mitochondria (the hemes in CIII and cytochrome *c*) and red blood cells (in hemoglobin). Fe-porphyrin in mitochondria can catalyze the hydrogenation of ROS, decreasing oxidative stress, and under hypoxia, it can reduce CO_2_ into CO—the signaling molecule inducing apoptosis in tumor cells [44]. In a hypoxic environment, red blood cells can capture H_2_ and scavenge ·OH in the blood circulation.

In summary, there is evidence that mitochondria may be the main intracellular target of H_2_, where Fe-porphyrin is able to catalytically hydrogenate ROS and donate electrons, compensating for electron leakage in the electron transfer chain [43] and rectifying electron flow in disordered states [41]. In this context, it should be mentioned that non-trivial quantum effects fundamentally contribute to the biological processes of charge separation. The efficient transfer of electrons between a donor and an acceptor in respiratory complexes and photosynthetic reaction centers are among the aspects of biology that cannot be accurately described by classical physics [45]. Quantum biology, as a field of biology that applies quantum theory to these aspects, can undoubtedly help to gain a deeper understanding of the mechanisms behind the mitochondria-targeted effects of molecular hydrogen.

CoQ_10_ is present in all cellular membranes, where it serves as an important lipophilic antioxidant. NAFLD is associated with the alteration of plasma and hepatic CoQ levels, but the data are conflicting [46]. In the model of NAFLD induced by high-fat diets in rats, Bravo et al. [47] reported increased levels of the reduced form of CoQ_9_ in plasma as an adaptive response to increased oxidative stress. In a mice model of NAFLD induced by a Western diet, authors Durand et al. [11] reported progressively increasing levels of both ubiquinone and semiquinone in liver mitochondria, with an increase in ubiquinone after 8 weeks and semiquinone after 16 weeks of Western diet feeding. In patients with NAFLD, decreased plasma levels of CoQ_10_ have been reported, which were negatively associated with body fat [48]. In our study, we found decreased levels of CoQ_10-TOTAL_ in patients with NAFLD, which is in accordance with the results reported by Yesilova et al. [48]. The 8-week treatment with HRW significantly increased the concentration of CoQ_10-TOTAL_ in platelets containing mitochondria [49], while in blood and plasma, the increase was non-significant (Figure 4).

Carotenoids act as antioxidants and can be metabolized to vitamin A, having immunomodulatory properties. Vitamins regulate cellular growth and metabolism. NAFLD may disturb the metabolism of fat-soluble vitamins (A, D, E, and K). The data on the concentration of lipid-soluble antioxidants in fatty liver disease are scarce. The knowledge about carotenoids in fatty liver has been reviewed recently [50]. Higher consumption of fruits containing carotenoids and higher concentrations of carotenoids in serum were found to be associated with a lower risk of NAFLD [51]. In patients with biopsy-proven NASH, circulating levels of α-tocopherol and carotenoids (β-carotene, α-carotene, lycopene, lutein, and zeaxanthin) were significantly decreased [52]. In our study, the levels of α-tocopherol in platelets and plasma and β-carotene in plasma were reduced in patients with NAFLD, which is in accordance with the study of Erhardt et al. [52]. After the 8-week treatment, the concentration of α-tocopherol in platelets increased and the concentration of β-carotene in plasma further decreased, similarly in both groups of patients. We suppose that the increase in the α-tocopherol concentration could be caused by a higher consumption of fats in the diet due to seasonal temperature changes (from autumn to winter). The decrease in the β-carotene concentration in both groups may come from a possible decreased consumption of fruits and vegetables in the winter time.

The lower levels of lipid-soluble antioxidants CoQ_10_, α-tocopherol, and β-carotene and the higher concentration of the marker of lipid peroxidation in our study reflect the presence of oxidative stress in patients with NAFLD. The concentration of plasma TBARS decreased after the 8-week treatment with HRW (Figure 6), indicating an antioxidant effect of HRW in patients with NAFLD. HRW may affect the antioxidant status by activating the nuclear erythroid 2-related factor 2 (Nrf2) pathway, which supports the production of innate antioxidants and a reduction of apoptosis and inflammation [53]. In the study of LeBaron et al. [29] on patients with metabolic syndrome, the concentrations of vitamins E and C increased and the plasma malondialdehyde level decreased after 24 weeks of therapy with HRW.

The results of our study showed the most pronounced effect of HRW treatment on the mitochondrial level: the concentration of CoQ_10_ increased in platelets, and the deteriorated parameters of platelet mitochondrial respiration improved. The effect of HRW on the CoQ_10_ concentration in platelets could be explained by a direct antioxidant effect of H_2_ in mitochondria: scavenging of ROS by H_2_ could prevent CoQ_10_ degradation. However, additional mechanisms may be involved. Our results support evidence that mitochondria are the primary target of H_2_ therapy. Additional and longer-term studies are needed to confirm the mitochondria-targeted effects of H_2_ therapy in patients with NAFLD.

## 4. Materials and Methods

### 4.1. Study Groups

A total of 30 patients with diagnosed NAFLD under treatment (13 males; 17 females) were included in the study. The inclusion criteria were steatosis according to USG (ultrasonography), overweight/obesity, increased liver enzymes alanine transaminase (ALT), aspartate aminotransferase (AST), and gamma-glutamyl transferase (GMT). Exclusion criteria were other serious health problems, such as acute inflammatory disease, rheumatological disease, cancer, decompensated diabetes, and heart, kidney, liver, or other organ failure. The patients filled out a questionnaire with questions about lifestyle risk factors.

The patients were randomly divided into 2 groups by a double-blinded method. The H_2_ group was formed by 17 patients (8 males; 9 females) with an average age of 52.6 ± 2.9 years. Patients in the H_2_ group received hydrogen-producing tablets with the ability to enrich regular water with molecular hydrogen (>4 mg/L H_2_) by the aqueous reaction between elemental magnesium and organic acids [54].

The placebo (P) group was formed by 13 patients (5 males and 8 females) with an average age of 53.2 ± 2.5 years. Patients in the placebo group received tablets that were similar in appearance and ingredients (magnesium carbonate, citric acid, sodium bicarbonate, Inulin, Kollidon 30, and sodium stearyl fumarate), where CO_2_ was produced instead of H_2_. Both types of tablets were donated by Drink HRW and Natural Wellness Now Health Products Inc. (Vancouver, BC, Canada).

All patients were instructed to dissolve one tablet in 330 mL of water and drink the enriched water produced immediately after dissolving the tablet three times per day for 8 weeks. During the study, patients continued the treatment with antihypertensive, antidiabetic, and hypolipidemic drugs without changes.

The control group consisted of 15 healthy volunteers (6 males and 9 females) with an average age of 51.3 ± 2.3 years.

### 4.2. Blood Collection

Blood samples were collected by venipuncture in two 9 mL VACUETTE^®^ tubes (Greiner Bio-One GmbH, Kremsmünster, Austria) with EDTA in the morning after overnight fasting at baseline and after the 8-week treatment.

### 4.3. Evaluation of Mitochondrial Function by High-Resolution Respirometry

Platelets were isolated from the whole blood as described previously [55] and counted on hematology analyzer Mindray BC-6200 (Shenzhen Mindray Bio-Medical Electronics Co., Ltd., Shenzhen, China). Mitochondrial bioenergetics in freshly isolated platelets was determined by high-resolution respirometry [56]. A total of 250 million cells were added into the 2 mL chamber of O2k-Respirometer (Oroboros Instruments GmbH, Innsbruck, Austria) with mitochondrial respiration medium MiR05 [56] and 20 mM Creatine at 37 °C. The data were collected with DatLab software (Oroboros Instruments GmbH, Innsbruck, Austria) using a data recording interval of 2 s [56]. The substrates, uncoupler, and inhibitors were added to the chamber following SUIT reference protocol 1 [27] under continuous stirring at 750 rpm. The order of titration was as follows: Dig—digitonin (0.36 µg/10^6^ cells); 1PM—CI-linked substrates pyruvate and malate (5 and 2 mM); 2D—adenosine diphosphate (ADP) (1 mM); 2D; c—cytochrome *c* (10 µM); 3U—uncoupler carbonyl cyanide 4-(trifluoromethoxy)phenylhydrazone (FCCP) (0.5 µM steps); 4G—CI-linked substrate glutamate (10 mM); 5S—CII-linked substrate succinate (10 mM); 6Rot—the inhibitor of CI rotenone (0.75 µM); 7Ama—the inhibitor of CIII antimycin A (2.5 µM). For evaluation of mitochondrial respiration, the respiratory rate after digitonin, representing residual oxygen consumption (ROX), was subtracted from all rates. The ROX-corrected respiratory rates after each titration were evaluated. The parameter *P-L* control efficiency was calculated as (2D—1PM)/2D [32].

### 4.4. Determination of Coenzyme Q_10_ Concentration

CoQ_10-TOTAL_ (ubiquinol and ubiquinone) in whole blood, plasma, and isolated platelets was determined using the HPLC method with UV detection [57], modified by authors [58,59]. For the oxidation of ubiquinol to ubiquinone, 100 µL of 1,4-benzoquinone (2 mg/mL double-distilled water—daily fresh) was added to 500 µL of blood or plasma sample and vortexed for 10 s. After 10 min of incubation at room temperature, 2 mL of the hexane/ethanol (5/2 *v*/*v*) mixture was added. The sample was shaken for 5 min and centrifuged at 1000× *g* for 5 min. The hexane layer was collected and the extraction procedure was repeated with 1 mL of the extraction mixture. Collected organic layers were evaporated under nitrogen at 50 °C. The residues were taken up in 99.9% ethanol and injected into reverse phase of HPLC column (SGX C18, 7 µm, Tessek Ltd., Strašnice, Czech Republic). Elution was performed with methanol/acetonitrile/ethanol (6/2/2 *v*/*v*/*v*) at a flow rate of 0.9 mL/min. The concentrations of CoQ_10-TOTAL_ were detected at 275 nm with a UV detector of CSW32 chromatographic station (DataApex Ltd., Prague, Czech Republic). The concentrations of CoQ_10-TOTAL_ were calculated in µmol/L using an external standard (Sigma-Aldrich, Saint-Louis, MO, USA).

The isolated platelets (150–250 million) were disintegrated with 500 µL of cold methanol [60]. Oxidation of ubiquinol to ubiquinone was performed with 1,4-benzoquinone, as described for plasma samples. The cell suspension was extracted with 2 mL of hexane by shaking for 5 min. After centrifugation, the organic layer was collected, evaporated, and processed as described above. Concentrations of CoQ_10-TOTAL_ were calculated in pmol/10^9^ cells.

### 4.5. Determination of TBARS

The parameter of oxidative stress, thiobarbituric acid reactive substances (TBARS), was determined by spectrophotometric method [61]. Plasma samples were mixed with ice-cold 76% trichloroacetic acid (TCA) and 1.07% thiobarbituric acid. After incubation at 100 °C for 30 min and cooling down, 90% TCA was added, and the sample was centrifuged at 2200× *g* for 15 min. The absorbance of supernatant was measured at 532 nm, and the concentration in μmol/L was calculated.

### 4.6. Data Analysis

The results in tables and bar graphs are shown as mean ± standard error of mean (sem). The graph with the correlation shows individual data points. Unpaired Student’s *t*-tests were applied to evaluate the difference between the parameters of the H_2_, P, and the control groups. Paired Student’s *t*-tests were used for evaluation of the difference in H_2_ and P groups between baseline values and the values after the 8-week treatment. *p*-values < 0.05 were considered statistically significant. Pearson correlation coefficient was evaluated with GraphPad Prism 6 for Windows (GraphPad Software, Boston, MA, USA).

## 5. Conclusions

In this study, we showed disturbances of platelet mitochondrial bioenergetics and CoQ_10_ levels in patients with NAFLD. Eight weeks of adjuvant therapy with molecular hydrogen (HRW) in patients with NAFLD improved platelet mitochondrial bioenergy function, increased CoQ_10_ concentrations in platelets, and reduced oxidative stress. We suppose that H_2_ may have protected CoQ_10_ from degradation or induced endogenous synthesis of CoQ_10_ in patients with NAFLD. A higher content of CoQ_10_ together with the direct effect of H_2_ as a rectifier of electron flow in the mitochondrial respiratory system, could improve mitochondrial electron transfer in platelets. Long-term supplementation with HRW could be a promising strategy for the acceleration of mitochondrial health and health recovery in patients with NAFLD. The application of H_2_ appears to be a new strategy for targeted therapy of mitochondrial disorders. Longer-term studies are needed to confirm the mitochondria-targeted effects of H_2_ therapy in patients with NAFLD. Studies that focus on the prevention of NAFLD and NASH by H_2_ application are warranted.

## Figures and Tables

**Table 1:** Anthropometric and biochemical parameters of groups of patients with NAFLD and healthy volunteers.

Parameter	Control	H_2_ before	H_2_ after	H_2_ before vs. Control	H_2_ after vs. H_2_ before	P before	P after	P before vs. Control	P after vs. P before	H_2_ before vs. P before
				*p*-value	*p*-value			*p*-value	*p*-value	*p*-value
Weight (kg)	73.9 ± 2.8	101.4 ± 3.7	100.3 ± 3.8	0.00001	ns	95.5 ± 4.0	96.0 ± 4.2	0.0001	ns	ns
BMI (kg/m^2^)	25.2 ± 0.9	35.5 ± 1.0	35.2 ± 1.1	0.00001	ns	32.8 ± 0.9	32.9 ± 0.8	0.00001	ns	ns
Waist (cm)		115.3 ± 2.4	113.4 ± 2.9		0.038	112.6 ± 2.6	110.3 ± 2.2		ns	ns
LYM (10^9^/L)	2.11 ± 0.16	2.35 ± 0.17	2.62 ± 0.25	ns	0.018	2.42 ± 0.19	2.56 ± 0.19	ns	ns	ns
ALT (μkat/L)	0.45 ± 0.07	0.76 ± 0.11	0.75 ± 0.07	0.033	ns	0.69 ± 0.09	0.58 ± 0.07	0.054	0.095	ns
AST (μkat/L)	0.41 ± 0.04	0.63 ± 0.10	0.55 ± 0.05	0.057	ns	0.57 ± 0.06	0.48 ± 0.04	0.034	0.056	ns
GMT (μkat/L)	0.38 ± 0.06	0.92 ± 0.18	0.95 ± 0.22	0.014	ns	0.64 ± 0.10	0.56 ± 0.06	0.037	ns	ns
ALP (μkat/L)	1.26 ± 0.10	1.28 ± 0.12	1.34 ± 0.13	ns	ns	1.07 ± 0.09	1.09 ± 0.10	ns	ns	ns
GLU (mmol/L)	5.13 ± 0.17	7.67 ± 0.71	7.73 ± 0.63	0.004	ns	6.75 ± 0.69	6. 25 ± 0.33	0.026	ns	ns
Albumin (g/L)	47.0 ± 0.7	42.4 ± 0.6	44.4 ± 0.5	0.00004	0.00003	43.2 ± 0.7	44.5 ± 0.8	0.001	0.009	ns
CHOL (mmol/L)	5.32 ± 0.27	4.65 ± 0.25	4.87 ± 0.24	0.079	ns	4.64 ± 0.23	4.86 ± 0.29	0.070	ns	ns
HDL-CH (mmol/L)	1.41 ± 0.13	1.11 ± 0.03	1.19 ± 0.03	0.026	0.00003	1.15 ± 0.07	1.19 ± 0.06	ns	ns	ns
TAG (mmol/L)	2.05 ± 0.49	1.92 ± 0.18	1.97 ± 0.21	ns	ns	1.92 ± 0.18	2.21 ± 0.22	ns	ns	ns

BMI—body mass index; LYM—the lymphocytes count in the blood; ALT—alanine transaminase; AST—aspartate transaminase; GMT—gamma-glutamyl transferase; ALP—alkaline phosphatase; GLU—glucose; CHOL—total cholesterol; HDL-CH—high-density lipoprotein (HDL) cholesterol; TAG—triglycerides. Control—the control group; H_2_ before, H_2_ after—the H_2_ group of patients with NAFLD at the beginning and the end of the study; P before, P after—the placebo group of patients with NAFLD at the beginning and the end of the study. The differences between H_2_ before, P before, and the control group, between H_2_ before and P before, and between H_2_ before and H_2_ after, and P before and P after groups are statistically evaluated. Values of *p* < 0.05 are considered statistically significant, values of *p* < 0.1 are shown, values of *p* > 0.1 are considered statistically non-significant and are replaced by the abbreviation ns (non-significant).

## Data Availability

The data is contained within the article.
